# Molecular Subtypes, microRNAs and Immunotherapy Response in Metastatic Colorectal Cancer

**DOI:** 10.3390/medicina60030397

**Published:** 2024-02-26

**Authors:** Alexandra Gherman, Dinu Bolundut, Radu Ecea, Loredana Balacescu, Sebastian Curcean, Constantin Dina, Ovidiu Balacescu, Calin Cainap

**Affiliations:** 1 10th Department of Medical Oncology, University of Medicine and Pharmacy “Iuliu Hatieganu”, 8 Victor Babes Street, 400012 Cluj-Napoca, Romania; allexandragherman@gmail.com (A.G.); calincainap2015@gmail.com (C.C.); 2 Department of Medical Oncology, The Oncology Institute “Prof. Dr. Ion Chiricuta”, 34-36 Republicii Street, 400015 Cluj-Napoca, Romania; dinubolundut@yahoo.ro (D.B.); radu.ecea@gmail.com (R.E.); 3 Department of Genetics, Genomics and Experimental Pathology, The Oncology Institute “Prof. Dr. Ion Chiricuta”, 400015 Cluj-Napoca, Romania; loredana_balacescu@yahoo.com; 4 10th Department of Radiation Oncology, University of Medicine and Pharmacy “Iuliu Hatieganu”, 8 Victor Babes Street, 400012 Cluj-Napoca, Romania; sebastian.curcean@gmail.com; 5 Department of Radiation Oncology, The Oncology Institute “Prof. Dr. Ion Chiricuta”, 34-36 Republicii Street, 400015 Cluj-Napoca, Romania; 6 Department of Anatomy, Faculty of Medicine, Ovidius University, 124 Mamaia Boulevard, 900527 Constanta, Romania

**Keywords:** CRC, molecular subtypes, tumor microenvironment, immune checkpoints, immunotherapy, microRNAs

## Abstract

Currently, only a limited set of molecular traits are utilized to direct treatment for metastatic CRC (mCRC). The molecular classification of CRC depicts tumor heterogeneity based on gene expression patterns and aids in comprehending the biological characteristics of tumor formation, growth and prognosis. Additionally, it assists physicians in tailoring the therapeutic approach. Microsatellite instability (MSI-H)/deficient mismatch repair proteins (MMRd) status has become a ubiquitous biomarker in solid tumors, caused by mutations or methylation of genes and, in turn, the accumulation of mutations and antigens that subsequently induce an immune response. Immune checkpoint inhibitors (ICI) have recently received approval for the treatment of mCRC with MSI-H/MMRd status. However, certain individuals experience either initial or acquired resistance. The tumor-programmed cell death ligand 1 (PD-L1) has been linked to the ability of CRC to evade the immune system and promote its growth. Through comprehensive research conducted via the PUBMED database, the objectives of this paper were to review the molecular characteristics linked to tumor response in metastatic CRC in light of improved patients’ outcomes following ICI therapies as seen in clinical trials and to identify particular microRNAs that can modulate the expression of specific oncoproteins, such as PD-L1, and disrupt the mechanisms that allow the immune system to be evaded.

## 1. Introduction

Globally, cancer represents the second most important cause of noncommunicable disease mortality after cardiovascular diseases, with an estimated 9.3 million people losing their lives to cancer each year [1]. Colorectal cancer (CRC) is the third most common cancer and the second cause of cancer-related death worldwide [2]. Approximately 25% of CRCs are diagnosed at a metastatic stage and an additional 20% of cases develop metachronous metastatic disease, which poses challenges in achieving tumor control and often leads to cancer-related fatalities. Approximately 65% of CRC cases arise sporadically, resulting from acquired genetic and epigenetic events induced by modifiable risk factors that trigger intestinal inflammation and alter the microbiota [3]. The inflammatory bowel diseases (IBD) are widely recognized as chronic inflammatory conditions that have a well-established association with malignancy [4].

Extensive research into the management of mCRC has established that a comprehensive, multidisciplinary “continuum-of-care” treatment approach is essential. This approach should incorporate surgical removal or local therapies of the primary tumor and metastases, whenever feasible, in addition to molecularly targeted therapies and chemotherapy [5]. Nevertheless, despite the therapeutic advances, the 5-year survival rate only reaches 12% in a metastatic setting, with a median overall survival (OS) of approximately 30 months [6], a fact that can be partially attributed to the scarcity of predictive biomarkers [7].

The identification and subsequent utilization of PD-1/PD-L1 and CTLA-4 in clinical settings have garnered significant interest in the field of immune checkpoint blockade (ICB) immunotherapy. However, numerous clinical studies have highlighted the existence of a substantial proportion of patients who exhibit de novo or acquired resistance [5]. With the exception of MSI status, no other validated predictive biomarkers exist for immunotherapy response.

Immunotherapy alone has been recommended for patients with MSI-H/MMRd mCRC, which account for approximately 5% of all mCRC patients, including those with Lynch syndrome. Pembrolizumab (anti-PD-1) is the recommended first-line treatment in these patients, both by National Comprehensive Cancer Network (NCCN) in the U.S. [8] and European Society for Medical Oncology (ESMO) in Europe [5], whereas the combination of Nivolumab (anti-PD-1)/Ipilimumab (anti-CTLA-4) is currently recommended only by the NCCN. In second-line setting, the NCCN also recommends Dostarlimab (anti-PD-1) or Nivolumab monotherapy, while both societies recommend Pembrolizumab or Nivolumab/Ipilimumab for individuals who have not yet received immunotherapy [5,8]. Despite the existence of ongoing clinical trials concerning immune strategies for microsatellite stable (MSS) mCRC, these approaches are still in their early stages of investigation and do not yet have validated biomarkers to assist in the selection of suitable patients or to guide combination protocols with targeted therapies or chemotherapy, respectively. Therefore, it becomes essential to identify effective predictive biomarkers for better patient selection and prognostic biomarkers for outcome assessments [9].

Consensus molecular subtypes (CMS) have been identified to classify the genetic and molecular alterations in patients with CRC. By conducting comprehensive transcriptional genome analysis, the CMS system allows for the classification of CRC patients into four distinct molecular subgroups with prognostic and therapeutic implications. CMS1 or the “immune MSI” molecular subtype exhibits features associated with favorable response to ICIs [10].

In this context, treatment selection in mCRC has garnered increased interest. Recently, there has been a surge of interest and significant advancements in the study of the relationship between microRNAs and immunotherapy. MicroRNAs are extensively studied for their putative prognostic, predictive, and, more recently, therapeutic roles in the treatment of malignancies [11]. Given the advent of immunotherapies utilized in clinical settings and the paucity of predictive biomarkers, this review focuses on clarifying the impact of the molecular and genetic features within the molecular subtypes, and the modulation of anti-tumor immunity by tumor-associated miRNAs.

## 2. Materials and Methods

This review presents current understanding of the molecular mechanisms that govern the CRC microenvironment, by focusing on specific molecular subtypes and dividing the tumors into immunologically “improved” or “poor”, with therapeutic implications. Considering the identification and subsequent utilization of PD-1/PD-L1 and CTLA-4 in clinical settings, we have summarized the main clinical trials that lead to immunotherapy implementation in clinical practice.

Furthermore, we investigated the role of microRNAs as epigenetic regulators of immune evasion, with focus on PD-L1 as direct target. In this regard, we conducted a systematic literature search on the PUBMED database, using Medical Subject Heading (MeSH) and keywords to find relevant papers published up to January 2023. Search terms were microRNAs, miRNAs, miRs, programmed death ligand 1, PD-L1, PD L1, CD 274, B7-H1, B7 H1, colon cancer, rectal cancer, CRC, and colon adenocarcinoma. In addition, we conducted a thorough examination of the references of selected studies to identify any more relevant publications. All relevant papers published in English were included: experimental studies performed on in vitro or in vivo models, systematic reviews and meta-analyses, clinical studies.

## 3. MicroRNAs—An Overview

MicroRNAs (miRNAs) are endogenous, small, non-coding RNAs that function as regulators of gene expression at a post-transcriptional level [12]. 

The potential applications of miRNAs in cancer diagnosis, prognosis and therapy have been highly anticipated since their discovery. Distinct miRNA profiles can be recognized for various types of tumors, hence potentially serving as phenotypic indicators that can be utilized in different fields of cancer management. 

Human cancer is characterized by dysregulated miRNA expression via a number of mechanisms, including aberrant transcriptional control of miRNAs, dysregulated epigenetic modifications, defects in miRNA biogenesis and amplification or deletion of miRNA genes. They are known to have the ability to act as either tumor-suppressor genes or oncogenes by targeting of genes implicated in tumor development and progression, or genes involved in the suppression of the cell cycle, respectively. The dysregulated miRNAs have been demonstrated to exert a significant impact on the fundamental characteristics of cancer [13]. Furthermore, miRNAs have the ability to influence both the innate and adaptive immune system responses by regulating the activities of key immune system components, including macrophages, natural killer (NK) cells, and dendritic cells (DCs) [14]. The reciprocal interplay between miRNAs and immune checkpoints has been elucidated as a means of regulating their expression, suggesting that miRNA-based targeted therapy holds promise in the treatment of cancer [15].

**Figure 1:**
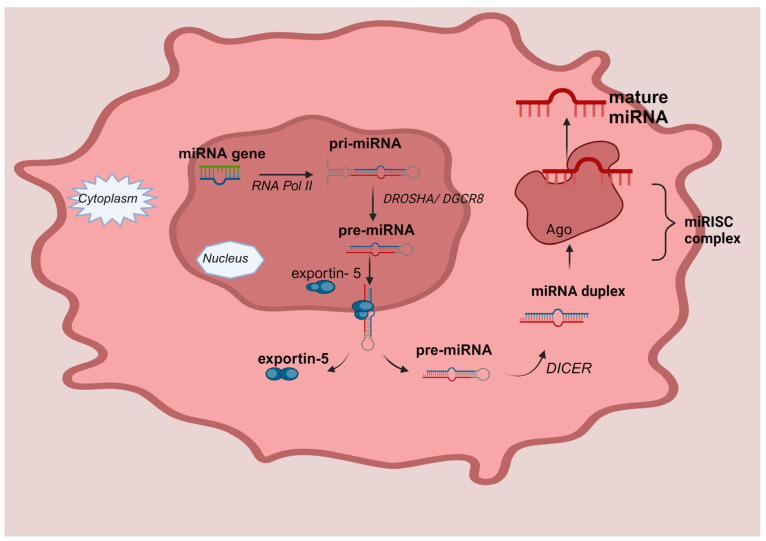
MicroRNA biogenesis. MiRNA genes are transcribed by RNA Polymerase II (RNA Pol II) called pri-mi-RNAs and cleaved into pre-miRNAs by Drosha (RNase III family) and its cofactor, the DiGeorge syndrome critical region eight (DGCR8 complex), in the nucleus. The pre-miRNA is transported to the cytoplasm through a mechanism that relies on Exportin5 and RanGTP, and it undergoes processing to generate the mature miRNA duplex. The circular conformation of the transcript is catalytically disrupted by the Dicer enzyme, leading to the formation of a double complementary strand molecule. Ultimately, one of the two strands, either the 5p or 3p strands, of the fully developed miRNA duplex is inserted into the Argonaute (AGO) proteins, which belong to the AGO family. This process results in the creation of a miRNA-induced silencing complex (miRISC).

Regarding their structure, miRNAs are short, non-coding molecules of RNA with a variable length, in the range of 17–25 nucleotides. As seen in Figure 1, the process of nuclear miRNA biogenesis commences with the production of primary miRNA transcripts, which often span a length of 300 to 1000 nucleotides. The production of these transcripts is facilitated by RNA polymerase II. Subsequently, in the nucleus begins the processing of the primary miRNA (pri-miRNA) through enzymatic cleavage facilitated by the Drosha enzyme (RNase III family), to form an intermediate, precursor miRNA with a hairpin structure, the pre-miRNA, of around 80–100 nucleotides. This cleavage event enables the pre-miRNA to be transported from the nucleus to the cytoplasm by the transporter protein esportin-5. Pre-miRNAs are further processed into the intracellular cytoplasm by the Dicer enzyme, which catalytically destroys the circular configuration of the transcript, resulting in the generation of a double complementary strand molecule. Ultimately, one of the two strands of the fully processed miRNA duplex is incorporated into the Argonaute (AGO) protein family, resulting in the formation of a miRNA-induced silencing complex (miRISC). The miRISC complex forms a binding interaction with certain target messenger RNAs (mRNAs), resulting in the induction of translational inhibition [12,13].

MiRNAs exhibit remarkable stability in bodily fluids, facilitating their extraction and quantification. Their established sensitivity and specificity render them highly ideal for biomarker research. MiRNAs are predominantly localized within intracellular compartments. However, a fraction of miRNAs is released into the extracellular environment by the secretion of lipid-enclosed vesicles called exosomes [16]. Extracellular miRNAs serve as chemical signaling molecules that facilitate intercellular communication [17].

A single miRNA can potentially target hundreds of mRNA molecules. In the majority of cases, miRNAs exhibit binding affinity towards the 3′ untranslated region (3′UTR) of their mRNA targets. Notably, these 3′UTR regions also encompass binding sites for several miRNAs, facilitating potential interactions among them through synergistic or competitive mechanisms. Presently, the human genome has been documented to have approximately 2000 microRNAs (miRNAs), which are responsible for regulating approximately 60% of the coding genes [18].

## 4. The Role of microRNAs in the Diagnosis, Prognosis, and Prediction of Response in CRC

MiRNAs possess several advantageous characteristics that render them clinically valuable as biomarkers: precise diagnostic value, steady availability in human fluids, and minimally invasive detection. According to recent studies, several miRNAs have emerged as potential biomarkers for the diagnosis, prognosis, and prediction of tumor responses in CRC. A great body of clinical research has identified tissue, stool, or circulating miRNAs or panels of miRNAs as of great potential in this regard. The main currently employed approaches for screening CRC include endoscopic methods (such as colonoscopy and flexible sigmoidoscopy), imaging techniques (such as computed tomographic colonography), and stool-based tests. However, non-invasive plasma tumor markers like carcinoembryonic antigen (CEA) or CA 19-9 lack the necessary sensitivity and specificity and are not recommended. Considering the invasiveness of the endoscopies, individual compliance with recommended screening procedures is low. In a novel blood-based test, the methylation status of septin9 showed a sensitivity of 68% and a specificity of 80% in diagnosing CRC, but this is not yet validated for systematic use worldwide [19]. Therefore, there is a significant need for minimally-invasive biomarkers in this context.

Extensive studies have investigated the diagnostic role of oncogenic miR-92a in CRC. The levels of miR-92a in the stool were reported to be involved in early diagnosis and the diagnosis of CRC, with a sensitivity of 71.6% and a specificity of 73.3% [20]. As shown by the authors, the levels of miRNA-92a in stool samples were lower in patients without malignancies compared to those with CRC. Nonetheless, as shown by other authors, the serum exosome levels were significantly higher in metastatic stages than in localized disease [21]. Conev and colleagues showed that the serum expression of miR-17, miR-21, miR-92 was higher in cases of disease recurrence [22]. A panel of six serum miRNAs, miR-21, let-7g, miR-31, miR-92a, miR-181b, miR-203 were reported to have a sensitivity of 96.4% and a specificity of 88.1% in diagnosing CRC, as reported by Wang et al. in their study [23]. The capacity of circulating miR-21 to diagnose CRC was also shown by other research studies [24,25]. Tissue levels of miR-429 were shown to be associated with CRC diagnosis, prognosis, and response prediction to first-line 5-fluorouracil (5-FU)-based chemotherapy. The level of expression of miR-429 was significantly higher in CRC compared to adjacent normal tissue, but also in the serum of CRC patients compared to healthy donors. Higher levels were associated with a higher TNM stage, worse OS, and non-responders to chemotherapy [26]. OncomiR-196b-5p, known to be involved in JAK/STAT3 signaling, was shown to be overexpressed in CRC primary tumors vs. adjacent healthy ones, but also in the serum exosomes of CRC patients vs. healthy donors, and was positively correlated with metastatic disease and lower OS; in vitro studies also correlated it with a lack of response to 5-FU [27]. Maintenance of cancer stemness by miR-196b-5p contributes to the chemoresistance of CRC cells via activating the STAT3 signaling pathway [28]. Another study demonstrated that CRC patients exhibited elevated levels of serum miR-135b, which was found to alleviate chemoresistance to oxaliplatin in mice [29]. miR-143 was shown to be downregulated in CRC tissues vs. healthy surrounding ones and also in plasma levels of CRC patients vs. healthy donors; more advanced stages of CRC had a lower expression of miR-143. In vitro studies showed that miR-143 could enhance chemosensitivity to oxaliplatin [30]. Low levels of miR-486-5p and miR-181a-5p in plasma exosomes, as hypoxia markers in advanced rectal cancer, were shown to be associated with the invasiveness of the primary tumor and lymph node involvement [31]. According to Chen et al., the expression of miR-100 appears to be downregulated in CRC tissues versus normal adjacent ones, and low miR-100 expression seems to be correlated with a higher TNM stage and a lower OS [32].

Regarding the potential of the miRNAs to predict the response to systemic therapies, several studies aimed to establish miRs’ potential of guiding treatment choice in CRC. The serum exosomal expression of miR-92a-3p, an oncogenic miR in CRC, was shown to be both diagnostic and predictive for response to 5-fluorouracil + oxaliplatin (FOLFOX) chemotherapy. Higher levels were associated with a lack of response [33]. Patients treated with capecitabine (CAPE) for stage IV CRC that exhibited low levels of miR-143 expression in the primary tumors had improved progression-free survival (PFS) [34]. miR-484 is a tumor suppressor in CRC, and high levels of expression in the plasma of patients with mCRC, together with low levels of miR-106a and miR-130b, were associated with a lack of response to chemotherapy in the study conducted by Li et al. [35]. Other research teams found an association between a low tissue expression level of miR-31-3p and a better response to anti-EGFR monoclonal antibodies [36]. High expression of miR-345 in the whole-blood was found to be associated with OS, PFS, and a lack of response to cetuximab + irinotecan in mCRC, as shown by Schou and team [37]. Low expression of miR-31-3p in RAS WT metastatic CRC patients was shown to be associated with a better response from cetuximab vs. bevacizumab in terms of survival parameters [38].

In our previous recently published research, we investigated the predictive value of exosomal plasma miRNAs for chemosensitivity in CRC cancer. Our results showed that significantly higher baseline levels of miR-92a-3p, miR-146a-5p, miR-221-3p, and miR-484 were expressed in non-responders vs. responders, and increased baseline miR-92a-3p and miR-221-3p predicted a lack of response to chemotherapy and lower OS [11].

## 5. Current State-of-the-Art Treatment in CRC

Standard treatment options in colon and rectal cancer, as recommended by the NCCN and the ESMO, are depicted below. Surgery is the mainstay of treatment in localized or loco-regionally advanced colon cancer, with adjuvant treatment added based on the TNM stage, according to the AJCC 8th edition [39] (Table S1 Supplementary File) and the presence of risk factors. High-risk features include major risk factors for recurrence (T4 and/or less than 12 resected lymph nodes) or minor risk factors, exclusive of those who are MSI-H (poorly differentiated histology, lympho-vascular space invasion (LVSI), bowel obstruction, perineural invasion, close or positive margins, high tumor budding, and preoperative CEA [40].

For MSI-H colon cancer stages 0–IIB, surgery is the recommended treatment. In stage IIC, the options following surgery include observation or adjuvant chemotherapy with either 3 months of CAPE + oxaliplatin (CAPOX) or 3–6 months of FOLFOX. In selected cases, a fluoropyrimidine (FP), such as CAPE or 5-FU, can be recommended for a duration of 6 months. Stage III colon cancer requires adjuvant chemotherapy, similar to stage IIC. Patients with MSS colon cancer stages 0–I undergo observation after surgery. In stage II A with no high-risk features, observation or a FP for 6 months are advised. If stage II A with high-risk features or stages IIB/IIC, either observation or adjuvant chemotherapy (a FP for 6 months, 3 months of CAPOX, or 6 months of FOLFOX) should follow surgery. For stage III disease, adjuvant chemotherapy is recommended [8,40].

High-risk features in rectal cancer are positive resection margins, LVSI, poorly differentiated tumors, and submucosal invasion to the lower third of the submucosa [41]. In clinical stage cT1N0M0, endoscopic submucosal resection or transanal local excision is recommended, while in cT1-2 N0 and cT3 N0, low-risk, high rectal tumors, transabdominal resection is the standard treatment. For cases with upfront surgery and high-risk pathological stage pT1 or pT2, chemoradiation is advised. For pT ≥ 3 and/or pN+ disease, adjuvant chemoradiation and chemotherapy are recommended for up to 6 months. If the clinical stage is T ≥ 3 and/or cN+ or an unresectable tumor, the choice of the initial therapy depends on the MSI/MMR status. MSS disease is treated by either long-term chemoradiation, or short-term radiotherapy, followed by 12–16 weeks of FOLFOX/CAPOX or FOLFIRINOX chemotherapy and then surgery or observation in case of a complete response. The chemotherapy can also be administered before the radiotherapy [41,42]. In MSI-H disease, the NCCN guidelines recommend neoadjuvant or definitive immunotherapy as the preferred choice of therapy with either Dostarlimab, Nivolumab, or Pembrolizumab. In cases of complete response at 6 months, surveillance is recommended; otherwise, the addition of long-course chemoradiation or short-course radiation, followed by transabdominal resection and further surveillance or chemotherapy doublet for up to 12–16 weeks. Another option is total neoadjuvant therapy, as described in MSS disease [42].

Metastatic CRC is treated according to the resectability of the primary tumor and metastases, the molecular profile, tumor sidedness, and the MSI/MMR status. In the case of metastatic rectal cancer with limited, resectable metastatic disease, the treatment for the rectal primary tumor follows the recommendations according to the T and N stages, as described in non-metastatic disease [5,42].

MMR proficient/MSS disease. In cases of resectable, synchronous liver-only and/or lung metastases, the standard treatment is synchronous or staged colectomy and local treatment for metastases, with either perioperative or adjuvant chemotherapy, for a total of 6 months. In case of potentially convertible metastatic disease, it is advisable to administer chemotherapy (FOLFOX/CAPEOX/FOLFIRI/FOLFIRINOX) and targeted therapies (bevacizumab or, in cases of KRAS/NRAS/BRAF wild-type and left-sided tumors, cetuximab/panitumumab), followed tumor response assessments every 2–3 months. If resectability is achieved, adjuvant chemotherapy ± targeted therapies are recommended. If the disease remains unresectable, clinicians should recommend systemic therapy +/− local therapies, where feasible. When the primary tumor and metastases are unresectable and imminent complications due to the colon tumor arise, colon resections, protective ostomy, bypass, or stenting are advised, in conjunction with systemic therapies. When the disease is considered unresectable, systemic therapies (chemotherapy plus targeted therapies, as above) represent the mainstay of treatment [5]. Second-line therapies depend on the chemotherapy and targeted therapies administered in the first line. Targeted therapies are added to standard chemotherapy according to tumor-sidedness and KRAS/NRAS/BRAF mutational status. If there is progression after an oxaliplatin regimen, it is advised to use irinotecan +/− 5-FU and an antiangiogenic agent such as bevacizumab (anti-VEGF-A), aflibercept (anti-VEGF-A and B, anti-placental growth factor), or ramucirumab (anti-VEGFR2) [8]. For cases of tumor progression after 1st line-irinotecan-based chemotherapy, it is advised to consider second line-fluoropyrimidine + oxaliplatin and either bevacizumab or an anti-EGFR, if KRAS/NRAS/BRAF WT. Second-line therapies also include biomarker-driven therapies, according to molecular alterations: BRAF V600E mutation (encorafenib + anti-EGFRs), HER-2 amplifications and RAS and BRAF WT (trastuzumab plus pertuzumab, lapatinib, or tucatinib), KRAS G12C mutations (sotorasib/adagrasib plus an anti-EGFR), RET gene fusion (selpercatinib), NTRK gene fusion (entrectinib, larotrectinib). Third-line and beyond options include fruquintinib (anti-VEGFR), regorafenib (multitarget tyrosine kinase inhibitor), trifluridine + tipiracil ± bevacizumab [5,8].

MMRd/MSI-H. If the disease is resectable stage IV, the options are either as in MSS disease, or neoadjuvant immunotherapy followed by surgery and local therapies for metastases. For synchronous unresectable disease, it is recommended to use ICIs, with tumor response assessments every 2–3 months. After first-line immunotherapy, further line therapies are based on the tumor location and molecular profile, similar to MSS disease [5,8].

## 6. Molecular Subtypes and Treatment Strategies for CRC

**Figure 2:**
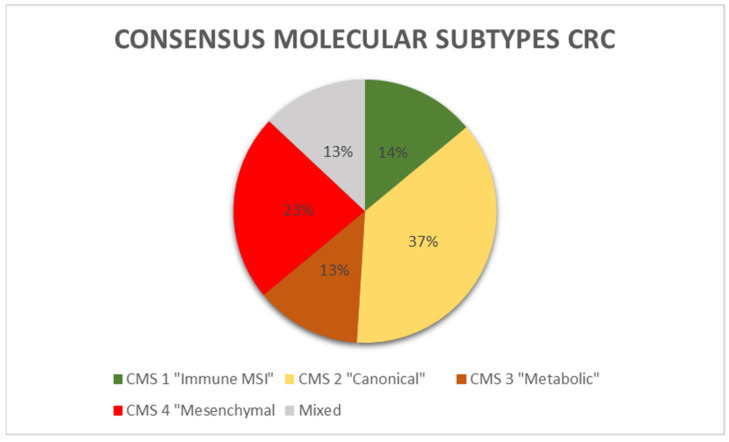
Consensus molecular subtypes distribution in CRC.

A recent proposal has put forth a molecular categorization of CRC with four distinct molecular subtypes, known as consensus molecular subtypes (CMS) [43]. Figure 2 depicts the percentage distribution of CMS in CRC.

According to the study performed by Guinney and collaborators [43], the defining features for each of the CMS classes are illustrated in Table 1.

The CMS1 subtype constitutes 14% of all CRCs and is characterized by hypermutated tumors with a significant immunological component and high levels of MSI. This particular subtype is commonly referred to as the “immune MSI” subtype. Genes linked to the activation of immune escape pathways and diffuse immune infiltrates, primarily composed of cytotoxic Th and T cells, and NK cell infiltration are expressed more frequently in CMS1 [44]. NK infiltration triggers the expression of immunological checkpoints, including PD-1, along with increased Th1 and cytotoxic T cell levels. CMS1 tumors overexpress PD-1 and CTLA-4. MSI-H cancers have a mutational rate that is 20 times higher than cancers with MSS. As a result, they produce a greater number of neoantigens. This high mutational oncogenic burden is associated with a deficiency in DNA mismatch repair (MMRd). The prevalence of BRAF mutations is highest in patients with CMS1. They were shown to be more common in women with less differentiated tumors and right-sided CRC. Immunotherapy using anti-PD-1 drugs may have a potential benefit for immunogenic MSI-H CRCs [45]. There is currently no strong data to support the usefulness of immunotherapy in MSS CRCs. Recent data indicate that conventional chemotherapeutics may induce the expression of PD.

37% of CRCs are CMS2, also known as the canonical subtype, which includes epithelial tumors that develop after the traditional, “canonical” route of carcinogenesis. These tumors have increased oncogene copy numbers and decreased tumor suppressor gene expression. Additionally, they exhibit significant chromosomal instability and prominent Wnt and MYC signaling. They occur more frequently in tumors of the left colon. The epithelial metabolic subtype, also known as CMS3, represents approximately 13% of all CRCs and is characterized by evident metabolic dysregulation. The occurrence of KRAS mutations is most commonly observed in tumors classified as CMS3. The CMS4 or mesenchymal subtype, which accounts for 23% of CRCs, is characterized by tumors that exhibit notable activation of TGF-β, stromal invasion, and angiogenesis. CMS4 tumors demonstrate increased expression of genes associated with the activation of MET and TGF-β, angiogenesis, matrix remodeling, infiltrates of stromal cells, and a greater presence of non-tumor cells in the microenvironment. CMS4 diagnoses occur at later stages of disease [43].

The efficacy of cetuximab or bevacizumab in addition to first-line 5-FU, folinic acid, and irinotecan (FOLFIRI) in patients with KRAS exon 2 wild-type mCRC was investigated in the FIRE-3 study (AIO KRK-0306), a randomized, open-label, phase 3 trial [10]. This study conducted an exploratory analysis on a specific subset of 438 patients from the intent-to-treat population in order to evaluate the objective response rates (ORR), OS, and PFS based on the four molecular subtypes. The patients were treated with a combination of FOLFIRI with either cetuximab or bevacizumab as their first-line treatment. The findings indicated that the molecular subtypes possessed substantial prognostic significance, regardless of the treatment administered. In the 315 RAS wild-type tumors, the observed frequencies were distributed as follows: CMS1 (12%), CMS2 (41%), CMS3 (11%), and CMS4 (34%). The CMS2 group exhibited the greatest median OS of 29 months, followed by the CMS4 group with 24.8 months, and the CMS3 group with 18.6 months. Lastly, the CMS1 group had the shortest OS, with a median of 15.9 months. The pattern seen for PFS was consistent with the aforementioned findings. In the cohort consisting entirely of RAS WT, it was shown that CMS2 tumors exhibited the highest ORR (78%) across both treatment groups, whereas CMS1 tumors displayed the lowest ORR (55%). The study findings indicate a notable improvement in ORR outcomes among patients who received a combination of chemotherapy and cetuximab in comparison to chemotherapy plus bevacizumab in CMS2 and CMS4. There was a notable increase in median OS and PFS among patients with all-RAS WT status who received cetuximab, compared to those who received bevacizumab, in patients classified as CMS4. However, for patients classified under CMS1 and CMS2, the outcomes were comparable between the two targeted treatments [10]. The CMS classification offers a more comprehensive understanding of its underlying biological characteristics; however, it currently does not have a direct influence on the decision-making process in clinical settings.

## 7. The Role of the Inflammatory Tumor Microenvironment in Immunotherapy Response

The immune system plays a dual role in CRC carcinogenesis. Intestinal inflammation, influenced by factors like diet, gut microbiota, and deregulated cytokines, chemokines, growth factors, and matrix-remodeling enzymes, contributes to the progression of CRC, the proliferation and survival of malignant cells, increases angiogenesis and metastasis, undermines adaptive immune responses, and modifies responses to chemotherapeutic agents. There is a pre-existing state of inflammation prior to the onset of malignancy. On the other hand, the occurrence of an oncogenic alteration triggers an inflammatory milieu. The presence of inflammation inside the tumor microenvironment (TME) elicits several responses that further promote tumorigenesis [46]. 

The communication between cells within the TME is mediated by cytokines and chemokines, the primary mediators of immune control [47]. Cytokines exert influence on various biological processes, including leukocyte recruitment, activation of immune cells, angiogenesis, and the turnover and differentiation of stem cells.

Tumor-infiltrating lymphocytes (TILs) consist of a heterogeneous population of immune cells, including T cells, B cells, NK cells, macrophages, and other innate cells, with T cells being the predominant subset. Ogino et al. indicated that increased levels of lymphocytic responses, such as Crohn’s-like lymphoid reactions, and TILs, were correlated with the prognosis of patients [48]. In their study, Edin et al. (2019) demonstrated a significant positive correlation between the presence of CD20+ B lymphocytes and CD8+ T cells, possibly due to a synergistic interaction [49]. In the study conducted by Koi and Carethers (2017), it was observed that diminished levels of T lymphocytes, specifically CD8+ and CD45RO+, as well as elevated levels of myeloid-derived suppressor cells (MDSC) and mast cells, are linked to an unfavorable immunological TME, consequently leading to decreased survival rates [50]. Additionally, high levels of Th17 cells and cancer-associated fibroblasts (CAFs) exhibiting immunosuppressive properties were also identified as indicators of poor survival [50].

NK cells play a crucial role in the first immune response, functioning as part of the innate immune system and facilitating programmed cell death. The presence of a diminished intra-tumoral population of NK cells has been found to be correlated with the phenomenon of immune evasion exhibited by the tumor [51]. DCs, recognized as professional antigen-presenting cells, play a crucial role in the immune system and are frequently hindered by the immunosuppressive TME. Disruption of DC activities by tumors plays a crucial role in immune evasion, tumor growth, the beginning of metastasis, and the development of resistance to treatment [52]. MDSCs are a population of immature myeloid progenitors that possess the ability to inhibit the function of T lymphocytes and NK cells, which are key components of the acquired immune response [53,54]. 

The quantity of mast cells within the tumor tissue is elevated in comparison to the neighboring healthy tissue. Activated mast cells are responsible for the release of growth-promoting factors and proangiogenic chemicals, leading to the activation of angiogenesis, extracellular matrix remodeling, tumor growth, and metastasis. Reduced levels of mast cells within the tumor were shown to be linked to decreased vascularity and improved survival outcomes in CRC [55]. Cancer-associated fibroblasts (CAFs) are very prevalent constituents of the TME [56]. They play a role in the onset of carcinogenesis, the release of growth factors, the process of angiogenesis, the migration of tumor cells, and tumor invasion and metastasis, as well as the formation of the extracellular matrix (ECM) and its related components. Tumors with elevated levels of fibroblast-activated proteins (FAPs) in the stroma demonstrate heightened aggressive traits [57]. Peritumoral myofibroblasts represent a significant cellular reservoir of cyclooxygenase-2 (COX-2) [58]. CAFs have been observed to generate many proinflammatory immunosuppressive factors and proangiogenic factors such as VEGFB, VEGFC, and PDGFC [59]. Francia et al. have referred to CAFs as the Trojan horse of resistance against antiangiogenic treatments [60]. 

To conclude, an improved immunological TME, also associated with a better prognosis in CRC, displays a high level and density of CD45RO+ CD8+ T lymphocytes and Th1 lymphocytes, and high expression of adhesion molecules, while a poor immunological TME often displays low levels of CD45RO+ and CD8+ T lymphocyte and high levels of MDSC, mast cells, Th17 as TILs and CAFs with immunosuppressive features [50,61]. The features of immunologically “hot”, or improved, and “cold”, or immunologically poor tumors, respectively, are shown in Table 2.

A great body of clinical research has provided evidence supporting the predictive significance of different indicators of inflammation in relation to cancer prognosis. The Immunoscore system [62] utilizes the measurement of tumor infiltration density by CD3+ T cells and CD8+ cells expressing a CD45RO+ phenotype. This particular phenotype has been linked to a more favorable prognosis, particularly when there is a higher infiltration of CD3+ and CD8+ cells in the central region of the tumor as opposed to its invasive borders. The aforementioned score exhibited a higher level of predictive accuracy for OS, compared to the MSI status in CCR. The analysis of systemic inflammation, which serves as an indicator of inflammation inside the TME, has also been the subject of scientific studies. The prognostic value of a modified Glasgow score (an elevated blood C-reactive protein coupled with hypoalbuminemia) has been confirmed in clinical trials involving more than 30,000 patients, although the predictive value for treatment response is still understudied [63,64].

Other studies have analyzed ways to mitigate inflammation as a means to both prevent and treat cancer while concurrently enhancing the chemosensitivity of tumors. Therapeutic approaches that aim to address inflammation primarily involve two strategies: obstructing the recruitment or depletion of pro-tumor inflammatory cells within the tumor and inhibiting the pro-tumor signaling pathways emitted by these inflammatory cells. Another approach involves redirecting antitumor inflammatory cells towards the TME [65]. 

The multifaceted nature of the inflammatory system’s involvement in cancer is further exemplified by the existence of T-cell-mediated antitumor immunity, which is triggered in suitable circumstances. Persistent inflammation can undermine the efficacy of this particular immune defense mechanism.

## 8. Immune Checkpoint Inhibition in CRC

Checkpoint inhibitors modify the immune response and improve endogenous anti-tumor action by inhibiting receptors on T cells or other cells that suppress T-cell function. T-cell activation is triggered by the recognition of antigens by the T-cell receptor. Co-stimulatory and inhibitory signals, often known as immunological checkpoints, regulate the extent of T-cell activation. In clinical trials, it has been demonstrated that ICIs are efficacious against immune evasion mechanisms. Regarding CRC, the cytotoxic T-lymphocyte-associated antigen 4 (CTLA-4), the programmed cell death protein 1 (PD-1), the lymphocyte-activation gene 3 (LAG3), the mucin domain-3-containing molecule 3 (TIM-3), and Indoleamine 2,3-dioxygenase (IDO) are recognized as significant inhibitory checkpoints that may play an important role in the growth and progression of tumors. Although a subset of patients has notable responses to ICIs, the majority of malignancies either display inherent resistance or acquire resistance following an initial positive response. Immunotherapy has been shown to be beneficial for immunogenic MSI-H CRCs; there is currently no strong data to support the usefulness of immunotherapy in MSS CRCs [66].

CRCs may become more responsive to immune checkpoint inhibitors if an appropriate immunological setting is established [67]. The efficacy of both conventional and targeted anticancer treatments is not limited to their direct cytotoxic effects. It also depends on their ability to (re)activate immune responses. Chemotherapy can enhance these reactions by boosting the immunogenicity of cancerous cells or by suppressing immunosuppressive mechanisms [68]. In preclinical studies on mice further validated on CRC patients, Dosset and collaborators [69] noticed an increase in the expression of PD-L1 and a significant infiltration of CD8 T cells in the TME of patients who underwent treatment with the FOLFOX regimen. In other studies, in addition to causing immunogenic cell death (ICD), oxaliplatin has been found to reduce the expression of programmed death ligand 2 (PD-L2), therefore reducing the immunosuppressive effects of both DCs and tumor cells [70].

### 8.1. CTLA-4 Immune Checkpoint

**Figure 3:**
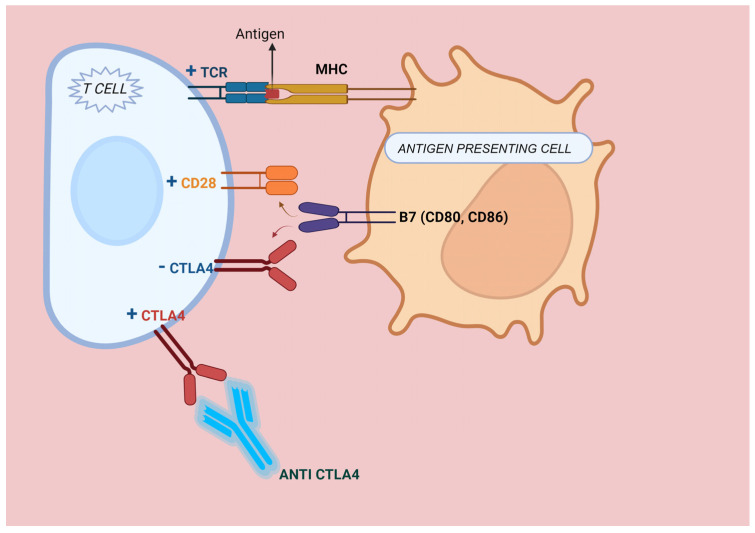
CTLA-4 Immune check-point blockade and T-cell activation. The tumor-associated antigen is presented by the antigen-presenting cell (APC) and recognized by the T-cell receptor (TCR) on the surface of the T-cell. T-cell activation necessitates a secondary stimulus, which is achieved through the interaction of B7 on the antigen-presenting cell (APC) with the CD28 receptor on the T-cell. CTLA-4 competes with CD28 for binding to B7 on APCs and leads to the suppression of T-cell activation. CTLA-4 antibodies inhibit CTLA-4 and reinstate T-cell activation.

Cytotoxic T-lymphocyte-associated protein 4 (CTLA-4) is a receptor that inhibits immune responses. It is a member of the CD28 immunoglobulin subfamily and is mostly found on T-cells. Figure 3 depicts the mechanism of CTLA-4 immune check-point blockade (ICB) and T-cell activation. Its ligands, CD80 (B7-1) and CD86 (B7-2) are commonly located on the surface of the antigen-presenting cells (APC). They have the ability to bind either CD28 or CTLA-4, leading to either a costimulatory or a co-inhibitory response, respectively. A competitive binding association exists between CTLA-4 and CD28, with CTLA-4 having a significant advantage because of its higher affinity for CD80/CD86 ligands [71].

### 8.2. PD-1 and PD-L1 Immune Checkpoints

**Figure 4:**
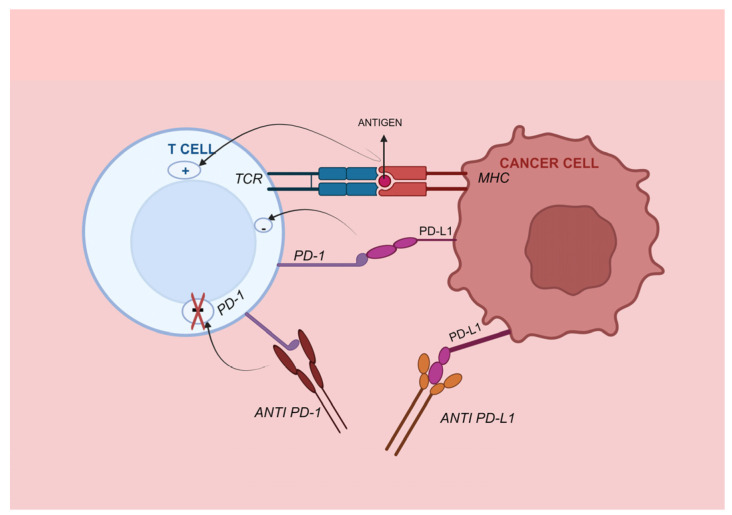
PD-1/PD-L1 immune checkpoint. PD-1 is a receptor found on the surface of immune cells that specifically interacts with its ligand, PD-L1. PD-L1 is found on antigen-presenting cells and cancer cells. When PD-1 is attached to PD-L1, it triggers anergy by weakening the signaling of the T cell receptor, and typically results in a decrease in T cell count. Blocking the PD-1 or PD-L1 pathway using a monoclonal antibody can enhance the immune response and hinder the growth of tumors. TCR, T cell receptor; PD-1, programmed death 1; PD-L1, programmed death ligand 1.

PD-1, also known as CD279, is a critical inhibitory checkpoint found on activated T cells. This transmembrane protein has two ligands: programmed cell death ligand 1 (PD-L1, CD274), which is present on activated B cells, T cells, monocytes, dendritic cells, vascular endothelial cells, and certain tumor cells; and PD-L2 or CD273, which is expressed on DCs, macrophages, mast cells, and specific B cell populations [72]. Figure 4 depicts the mechanism of PD-1/PD-L1 ICB and T-cell activation. A binding interaction between PD-1 and its primary ligand, PD-L1, results in the inhibition of T cell activation, cytokine release and cytotoxicity. Additionally, this interaction induces exhaustion and apoptosis of tumor-specific T cells; therefore, it regulates immunological responses and enables tumor cells to evade immune surveillance [73]. In a recent study, PD-L1 expression was more prevalent in liver and lung metastatic foci compared to the primary tumor [74].

The overexpression of the PD-L1 molecule within the TME impairs the immune response in various types of cancers. PD-L1 expression is also influenced by intrinsic carcinogenic pathways. Enhanced activity of the STAT3 transcription factors [75] and overactivation of intracellular signaling cascades involving the MAPK and PI3K-Akt pathways, all contribute to elevated PD-L1 expression on the cell membranes of malignant tumor cells [76,77]. Proinflammatory mediators, including interferon-γ and interleukin-6, increase the synthesis of PD-L1 [78]. The interaction between IFN-γ and its receptor triggers the activation of JAK1 and JAK2, resulting in the activation of the IFN-γ receptor [79]. Consequently, STAT1, a transcription factor located in the cytoplasm that has significant impacts on the development of tumors, becomes active, resulting in an induction of PD-L1 expression [80]. The overexpression of IL-6 can enhance the expression of PD-L1 in malignancies through the JAK/STAT3 signaling pathway [81].

Recent findings in non-small cell lung cancer (NSCLC) indicate that EGFR gene mutations directly correlate with the increased expression of PD-L1 [82,83]. EGFR activation is linked to excessive activation of the PD-1/PD-L1 pathway, possibly via IL-6/JAK/STAT3 pathway, resulting in an elevated likelihood of tumor immune evasion [84]. Nevertheless, the underlying mechanism behind this and its applicability in CRC remain unclear.

Higher levels of PD-L1 expression in tumor tissues theoretically correlate with an improved response to ICI treatment [85]. Over 50% of colon cancer patients [86] exhibit positive PD-L1 expression (10% cut-off). PD-L1 expression alone is insufficient for accurately predicting the response to immunotherapy in colon cancer. There are multiple factors that restrict the use of PD-L1 expression as a predictive biomarker, such as the variation in PD-L1 expression within the tumor itself, the inconsistency in PD-L1 expression between primary tumors and metastases, and the absence of standardized criteria for evaluating PD-L1 expression [87]. Nonetheless, both tumor cells and immune cells have the ability to express PD-L1. Therefore, it is necessary to establish distinct clarification on the individual impact of PD-L1 expression by tumor cells and PD-L1 expression by lymphocytes on the efficacy of immunotherapy [87].

Current research focuses on identifying composite scores to predict response to immunotherapy in CRC, encompassing different variables, such as the expression of PD-L1 and the percentage of extracellular mucin (CPM score) [88], and other immune biomarkers from the TME.

### 8.3. Immunotherapy in Clinical Practice in mCRC MSI-H and Emerging Strategies in MSS Disease

In clinical practice, MSI-H/MMRd has been associated with response to immunotherapy, particularly PD-L1/PD-1 inhibitors, and resistance to chemotherapy [89]. In CRC, MSI-H/MMRd is reported as between 4–5% in the metastatic setting, as opposed to 12–20% in stage I-III disease [9]. Table 3 summarizes pivotal clinical trials that led to the implementation of immunotherapy in clinical practice and other relevant, phase 2–3 clinical trials with anti-PD-1, anti-PD-L1 and anti-CTLA4 immune checkpoint inhibitors. 

The pivotal study supporting the use of immunotherapy in MSI-H mCRC is the randomized phase III trial, KEYNOTE-177, comparing pembrolizumab to standard chemotherapy regimens (FOLFOX/FOLFIRI +/− Cetuximab/Bevacizumab) in the first line setting. The study demonstrated a benefit in PFS: 16.5 m vs. 8.2 m, but no significant benefit in OS, possibly due to a high rate of crossover, at approximately 60%. A tendency towards OS improvement has been reported, with an OS rate of 54.8% vs. 44.2% at 5 years with a HR of 0.73 [90]. Consequently, pembrolizumab represents the standard of care for first-line treatment in mCRC MSI/MMRd according to the ESMO and NCCN guidelines [5,8].

The phase II Checkmate 142 trial reported on the benefit of adding a CTLA-4 inhibitor (ipilimumab) to a PD-1 inhibitor (nivolumab) in the first-line setting with an ORR of 69%, and a median PFS and median OS not reached at 2 years of follow-up [91]. The Checkmate 8 HW phase III confirmatory trial is ongoing [104]. Presently, Nivolumab–Ipilimumab is recommended in the first line setting by the NCCN guidelines. Other options recommended by the NCCN guidelines based on phase I/II clinical trials in the second line setting include nivolumab and dostarlimab [8].

In the second- or third-line setting, there are multiple phase I/II clinical trials reporting on the efficacy of PD-L1/PD-1 inhibitors: pembrolizumab, dostarlimab, nivolumab, and avelumab, with similar results, with an ORR between 30–38% [86,96,105,106]. The phase II Checkmate 142 trial tested the PD-1 and CTLA4 dual blockade in pre-treated patients, reporting a ORR of 55%, mPFS not reached [107]. The ESMO guidelines recommend pembrolizumab or nivolumab–ipilimumab in the second-line setting in immune checkpoint inhibitor naive patients, while the NCCN guidelines maintain the same recommendation as in the first-line setting [5,8]. 

Contrary to MSI, in MSS mCRC, immunotherapy is purely exploratory due to lack of response and efficacy. TMB-high or PD-L1 expression do not correlate with responsiveness to immunotherapy [108]. There are multiple trials combining immunotherapy with either new drugs or standard treatment in this setting.

The most common strategies include combining antiangiogenic drugs with checkpoint inhibitors, with mixed results in a phase I/II trial and a negative phase III trial [109], as well as combinations with chemotherapy. The AtezoTRIBE phase II, randomized trial, compared FOLFOXIRI + bevacizumab with or without atezolizumab, reporting a PFS benefit in the intention-to-treat population, statistically insignificant, but in the MSS population [94]. In a retrospective analysis of the AtezoTRIBE population, Immunoscore and Immunoscore-immune checkpoint correlated with response to immunotherapy, but not PD-L1 expression or TILs [110]. The MAYA trial proposed priming by temozolomide in MSS and MGMT-silenced disease, before introducing immunotherapy combination. The trial reported an ORR 45% and mPFS of 7 months in patients not progressing on temozolomide [111]. 

In conclusion, immunotherapy has become the current standard of care in mCRC MSI/MMRd, and current research focuses on surpassing resistance to immune checkpoint inhibitors in this setting, as well as identifying new biomarkers in the MSS population and novel immune-sensitizing combinations.

## 9. microRNAs on PD-1/PD-L1 Immune Checkpoint in CRC

MiRNAs can exert a role in regulating the anti-tumor activity of immune cells that have infiltrated the tumor. Several miRNAs also have a crucial role in modulating pro-inflammatory and anti-inflammatory pathways, namely those involving Toll-like receptors (TLRs), nuclear factor kappa B (NF-κB), and transforming growth factor beta (TGF-β) [112]. Activation of innate and adaptive immune cells, such as macrophages, neutrophils, T cells, B cells, and others, is also facilitated by a subset of additional miRNAs [113]. Moreover, miRNAs present in tumors can be transmitted from tumor cells to nearby immune cells, resulting in alterations to the immune response. Our objective was to ascertain miRNAs that regulate immune evasion and post transcriptionally modulate the expression of PD-L1 on tumor cells. 

The study conducted by Zhao et al. [15] examined the impact of miR-138-5p on the development of CRC. MiR-138-5p was often downregulated in CRC tissues and was linked to advanced clinical stage, lymph node metastases, and a poor OS rate. In vitro, miR-138-5p slowed down the growth of CRC cell lines and partially stopped them from entering the S-phase by decreasing PD-L1. They also used in situ hybridization (ISH), immunohistochemistry (IHC), and quantitative reverse transcription polymerase chain reaction (qRT-PCR) to look at the levels of PD-L1 and miR-138-5p in CRC tissues. They detected an inverse association between PD-L1 and miR-138-5p levels in tumor tissues. Patients exhibiting elevated levels of PD-L1 expression demonstrated a heightened likelihood of mortality (*p* = 0.0024), suggesting that PD-L1 expression may serve as a prognostic indicator for CRC.

In the research performed by Jiang et al. [114], the expression of miR-140-3p was markedly reduced in both CRC tissues and cell lines, and there was an increased expression of PD-L1. Upregulation of miR-140-3p suppressed tumor growth and infiltration and triggered the programmed cell death of CRC cells. Researchers identified PD-L1 as a potential target gene of miR-140-3p. Inhibiting PD-L1 expression in CRC cells led to biological responses that were similar to those seen after miR-140-3p mimics were used to treat the cells. Increasing PD-L1 expression again decreased miR-140-3p’s inhibitory effect on CRC cells by a small amount. 

Chen et al. [115] showed that the expression of miR-93-5p was reduced in CRC tissues, while the expression of PD-L1 was increased. miR-93-5p expression was elevated in PD-L1 negative patients. The expression levels of miR-93-5p and PD-L1 were correlated with the degree of differentiation, the TNM staging system and the prognosis. In addition, anti-PD-L1 increased the levels of interleukin-2 (IL-2), tumor necrosis factor-α (TNF-α), and interferon γ (IFN-γ) in the coculture of T cells with CRC cells, while decreasing the levels of IL-1β, IL-10, and TGF-β. Nevertheless, the effects of miR-93-5p suppression largely counteracted these alterations.

Roshani et al. [116] showed that the expression of miR-124 is considerably reduced in CRC tissues compared to adjacent normal samples (*p* < 0.0001). Transfecting HT29 and SW480 cells with miR-124 mimics resulted in a significant decrease in PD-L1 mRNA and cell surface expression of PD-L1, and it also inhibited Tregs in coculture models. The overexpression of miR-124 resulted in a decrease in CRC cell proliferation and arrested the cell cycle at the G1 phase by reducing the expression of c-Myc. They also demonstrated that miR-124 suppresses STAT3 signaling in CRC cells.

The team conducted by Ashizawa [117] showed that the downregulation of miR-148a-3p regulates PD-L1 expression on tumor cells, resulting in immune suppression in CRC. Through the analysis of various cohorts of CRC, including TCGA data, a microarray dataset (n = 148), and FFPE samples (n = 395), they observed a reduction in the expression of miR-148a-3p in MMRd/MSI-H tumors, inversely correlated with the levels of PD-L1. They also proved that miR-148a-3p attaches specifically to the 3′-UTR region of PD-L1. This lowers the amount of PD-L1 in HCT116 and SW837 cell lines.

Martinez-Ciarpaglini and colleagues [118] conducted an analysis on a set of 125 samples of colon cancer. The expression of PD-L1 was markedly increased in the budding regions at the invasive tumor front, and its levels were positively associated with a mesenchymal transition profile. They also discovered a significant decrease in miR-200a, miR-200b, and miR-200c levels in the budding areas located at the invasive front of the tumor. This decrease was found to be associated with a poorer survival outcome in early-stage disease, as determined in multivariate analysis. Their results offer evidence of the impact of mesenchymal transition on immunological resistance facilitated by PD-L1 overexpression.

In their research, Jin et al. [119] showed that miR-382-3p exhibited the ability to reduce the levels of PD-L1 in HCT116 and Caco-2 cells. The presence of MiR-382-3p has been linked to a reduction in tumor growth and an increase in programmed cell death in CRC cells [50]. The expression of miR-382-3p was significantly reduced in CRC tissues, while its increased expression is linked to better OS.

Chen and colleagues [120] performed a study to assess the clinical significance of PD-L1 in CRC. The study included a group of 240 CRC patients from The Cancer Genome Atlas (TCGA), as well as a group of 40 CRC pair-matched samples. The expression level of PD-L1 was elevated in CRC samples (n  =  40) in comparison to pair-matched neighboring normal tissues. The increased expression of PD-L1 was found to be associated with a worse outcome in individuals with advanced stage CRC. The level of miR-191-5p showed a negative relationship with the expression of PD-L1 and served as an independent prognostic indicator for OS in patients with CRC. Reduced miR-191-5p expression was linked to OS and disease recurrence. Thus, PD-L1 could serve as an indicator of worse prognosis and is inversely correlated with the expression of miR-191-5p in CRC patients. 

Liu et al. [121] conducted their study based on prior findings that demonstrated the role of IL-17A, in conjunction with T-helper 17 cells, in promoting tumor growth, angiogenesis, inhibition of the immune system through regulatory T cells, and resistance to antitumor immunity. Through experiments conducted on MSS CRC cell lines, tissue samples, and in vivo research using mouse models, it was demonstrated that miR-15b-5p reduced the expression of PD-L1 and increased the susceptibility to anti-PD-L1 treatment. IL-17A induced elevated PD-L1 expression in CRC cells by modulating the P65/NRF1/miR-15b-5p pathway and enhanced resistance to anti-PD-1 treatment. The efficacy of anti-PD-1 therapy increased in MSS CRC murine models by blocking IL-17A, making it a potential therapeutic target.

The expression of miR-22 is reduced in CRC and has a role in increasing the sensitivity of colon cancer cells to 5-FU chemotherapy [122]. Tian et al. [123] conducted a study examining the function of histone methyltransferase (SETDB1) in immune evasion in CRC (CRC) and its association with PD-L1 via miR-22. Increased levels of SETDB1 were correlated with higher levels of PD-L1 expression. SETDB1 decreased the expression of miR-22, whereas miR-22 reduced the level of PD-L1 through BATF3. Collectively, SETDB1 had the potential to stimulate the BATF3/PD-L1 pathway by suppressing miR-22, hence facilitating immune evasion in CRC. Suppression of SETDB1 had increased the ability of T cells to kill tumor cells by modulating the FOSB/miR-22/BATF3/PD-L1 pathway, hence impeding the formation of CRC tumors in mice.

Circular RNAs were long thought to be non-functional RNAs, but new research has revealed their involvement in a number of pathogenic processes, including tumor development. MiR-497 is reported to be downregulated in CRC. Circular RNA hsa_circ_0136666, another non-coding RNA, has also been shown to be involved in the development of CRC. The role of hsa_circ_0136666 in immune evasion through the miR-497/PD-L1 pathway was studied by Xu and colleagues [124]. According to their findings, there was an increased expression of PD-L1 and hsa_circ_0136666 in CRC cells. It was shown that Hsa_circ_0136666 directly targets miR-497, which in turn binds to the 3′UTR of PD-L1 to control it. By suppressing miR-497 levels, Hsa_circ_0136666 increased PD-L1 expression in CRC, thereby stimulating the activation of regulatory T cells and facilitating the tumor’s evasion of the immune system. 

It has been shown that miR-214 inhibits the growth of several malignancies, including CRC. The research conducted by Yang et al. [125], performed on CRC cell cultures and xenografts on mice, aimed to shed light on the fundamental process of circEIF3K-regulated CRC carcinogenesis and metastasis. They discovered that exosomal circEIF3K might be secreted in response to hypoxia and demonstrated the significance of PD-L1 as a miR-214 target in CRC. A summary of miRNAs targeting PD-1/PD-L1 immune checkpoint in CRC is presented in Table 4.

## 10. Conclusions and Future Directions

Despite significant improvements in the therapeutic landscape of mCRC, the prognosis remains dismal, with 5-year survival rates not surpassing 12%. The correlation between inflammation and the development of CRC is widely recognized and has been substantiated by a great body of evidence from clinical, pharmacological, immunological, and translational research over the past decades. Pembrolizumab was the first ICI approved by the Food and Drug Administration on 29 June 2020, for the initial treatment of patients diagnosed with unresectable or metastatic microsatellite instability-high (MSI-H) or mismatch repair deficient (MMRd) CRC. Due to the relatively recent advent of ICIs in the treatment arsenal, insufficient clinical and fundamental research data exist on predictive biomarkers for tumor response in mCRC, specifically beyond the MSI-H status. Highlighting the importance of the TME, the inflammation contributes to the proliferation and survival of malignant cells, increases angiogenesis and metastasis, undermines adaptive immune responses and modifies responses to chemotherapeutic agents.

A great body of clinical and translational research has focused on identifying not only specific features of the TME, but also, genetic and epigenetic alterations that could provide a better understanding of differences regarding treatment outcomes in mCRC patients. The emergence of the ICIs in the treatment of MSI-H/MMR-D mCRC has significantly changed the therapeutic landscape. Unfortunately, there is no other validated predictive biomarker for response to immunotherapy and, even among the 5% of mCRC with MSI-H/MMR-D disease, there are patients who exhibit de novo or acquired resistance, an aspect that underscores once more the disease heterogeneity. The CMS approach enables the classification of CRC patients into four unique molecular subgroups with prognostic and therapeutic implications through the use of thorough transcriptional genome analysis. The CMS1, also known as the “immune MSI” molecular subtype, displays characteristics that are linked to a positive response to immunological checkpoint inhibitors and chemotherapy resistance. Performing molecular profiling on patients with MSI-H/MMR-D CRC could help identify genetic changes that may elucidate the resistance mechanism and offer guidance for enrolling patients in clinical trials. 

Presently, there is insufficient research focused on alterations of tissue or circulating miRNAs expression levels to reflect the changes in the TME during and after treatment with ICIs. More research into how tumor miRNA profiles change after ICB treatment could help understand the mechanisms of resistance patterns and find new ways to address these into clinical practice. TME composition is dynamic and undergoes significant changes throughout ICB therapies. Studying the impact of various immunotherapies on the immune TME will provide more knowledge in the identification of predictive biomarkers and combination treatment approaches for better tumor control. Response to ICB is influenced by the composition of the TME. Pro-tumor immune cells, including MDSCs, Tregs, and TAMs, are crucial in developing an immunosuppressive microenvironment and hindering anti-tumor immune responses [126]. Nonetheless, longitudinal research conducted on melanoma patients treated with ICB showed, through repeated biopsies, that variations in the TME composition between responders and non-responders were more pronounced following two or three administrations of anti-PD-1 than at baseline [127,128]. The disparities were seen in the densities of CD4+ or CD8+ T cells between the two timepoints, and also in the expression of PD-1 or PD-L1. TCR repertoire clonality of responders was also found to be enhanced during therapy [127]. Furthermore, individuals who responded to ICIs displayed an elevated level of LAG3, a T-cell exhaustion marker, following the initiation of immunotherapy [127]. As shown by another research study in advanced melanoma, before and after initiation of nivolumab treatment, there was an increase in the numbers of CD8+ T cells and NK cells in responders vs. non-responders, as well as a decrease in macrophage infiltration [129]. In hematological malignancies, a disruption of the PD-1 pathway was shown to restore the function of CAR-T cells, and CAR-T cells may upregulate the expression of PD-1 signaling [130]. In an experimental study in experimental pancreatic cancer on mice, the combination of anti-PD-1 with anti-CTLA-4 resulted in higher T cell infiltration and tumor response, while blocking CSF-1/CSF-1R resulted in elevated PD-1/PD-L1 expression on TAMs and increased CTLA-4 expression on CD8+ T cells [131]. 

The discovery of miRNAs has generated great expectations for their potential applications in cancer detection, prognosis and therapy. Different types of tumors can be identified by their unique miRNA profiles, which can be used as indicators of their characteristics. Considering the fact that miRNA expressions can be manipulated to regulate the tumor phenotype, current research also focuses on utilizing miRNAs to develop cancer treatments, by either suppressing oncogenic miRNAs or restoring tumor suppressor deficient miRNAs, considering their role as regulators of protein-coding genes. Multiple therapeutic approaches exist that involve the manipulation of miRNA, and there are various methods for utilizing miRNA therapies. However, most of these treatments have only been confirmed through in vitro and in vivo research.

Considering their very high stability in tissue, including FFPE samples, miRNAs represent important biomarkers for managing cancer patients. Nevertheless, much research has focused over the past years on finding blood biomarkers that could improve the management of CRC patients, but there is not yet a clinical practice miRNA-based biomarker. Even if research data had highlighted the role of miRNAs in early prediction, prognosis, and treatment response in CRC, their translation to clinical practice must wait until the results are validated on large groups of patients in inter-centric studies. Moreover, to be able to use miRNA evaluation as a biomarker in managing CRC patients, a quantitative and individual evaluation method for miRNAs should be validated. Such a method could use digital PCR (ddPCR), which could make an accurate transfer from translational to clinical research, maintaining similar conditions and increasing accuracy, even between different laboratories.

To conclude, integrative analyses of DNA mutations, immune features of the TME, molecular subtypes and epigenetic traits, including miRNAs as modulators of the tumor phenotype, could have the potential to enhance our comprehension and stimulate forthcoming research on predicting the effectiveness of systemic treatments.

## Figures and Tables

**Table 1:** Consensus molecular subtypes (CMSs) in CRC. MSI-microsatellite instability; CIMP-CpG island methylator phenotype; SCNA—somatic copy number alteration; MSS—microsatellite stable; RFS-relapse-free survival.

CMS Class	Frequency	Tumor Location	Molecular Features	Immune Phenotype	Prognosis
CMS 1 “Immune MSI”	14%	77% right colon 20% left colon 3% rectum	MSI—high CIMP—high Hypermutated SCNA class—Low KRAS wt, NRAS wt BRAF m TP53 wt	Immune activation and infiltration	Worse RFS
CMS 2 “Canonical”	37%	59% left colon 23% right colon 18% rectum	MSS CIMP—negative SCNA class—high KRAS wt NRAS wt BRAF wt TP 53 m	Wnt and MYC activation Poorly immunogenic	Better RFS
CMS 3 “Metabolic”	13%	51% right colon 34% left colon 15% rectum	MSS CIMP—negative KRAS m NRAS wt BRAF wt TP53 wt	Metabolic deregulation Poorly immunogenic	
CMS 4 “Mesenchymal”	23%	47% left colon 35% right colon 18% rectum	MSS CIMP—negative SCNA class high KRAS wt NRAS wt BRAF wt	Stromal infiltration TGF β activator Angiogenesis	Worse RFS and OS

**Table 2:** Structure of immunologically “hot” and “cold” tumors. MDSC, meloid-derived suppressor cells; CAFs, cancer associated fibroblasts; NK, natural killer; DC, dendritic cells.

Improved Immunological TME	Poor Immunological TME
High inflammatory signature	Low inflammatory signature
High Immunoscore	Low Immunoscore
High levels of CD45RO+ CD8+ T cell, cytotoxic T cell infiltration, NK cells	Absent intra-tumoral CD8+ T cells, Low levels of NK cells
High levels of DCs	High levels of MDSC
High expression of adhesion molecules	High levels of mast cells
Immune Checkpoint activation	High levels of CAFs
	High levels of tumor infiltrating Th17 cells

**Table 3:** Clinical trials of immune checkpoint inhibitors in metastatic CRC.

Study	Type of Study	Status	Population	Setting	Intervention	Patients	Objectives	Results	Ref.
KEYNOTE-177 NCT02563002	phase III, randomized, open-label	completed	mCRC dMMR/MSI-H	1st line	pembrolizumab vs. chemotherapy +/− bevacizumab or cetuximab	N = 307	primary—PFS, OS; secondary—ORR	mPFS 16.5 vs. 8.2 m; ORR 43.8% vs. 33.1% mF-up of 44.5 m: mOS NR vs. 36.7 m G ≥ 3 AE 22% vs. 66%	[90]
CheckMate-142 NCT02060188	phase II	active, not recruiting	mCRC dMMR/MSI-H	1st line (cohort 3)	NIVO 3 mg/kg Q2W + IPI 1 mg/kg Q6W	N = 45	primary—ORR secondary—PFS, OS	5-year F-up: ORR 71% at 48 months: PFS = 51%, OS = 72%	[91]
COMMIT NCT02997228	phase 3, open-label, randomized	recruiting	mCRC dMMR/MSI-H	1st line	Arm I (control) + Bevacizumab Arm II Atezolizumab Arm III Atezolizumab + Bevacizumab + mFOLFOX6	estimated N = 241	Primary PFS; Secondary: OS, ORR safety, DCR, DOR	Ongoing, not reported	[92]
SEAMARK NCT05217446	Phase 2, randomized	recruiting	mCRC-dMMR/MSI-H BRAFm	1st line	Arm A: Encorafenib + Cetuximab + Pembrolizumab, cetuximab Arm B (control): Pembrolizumab	N = 104	Primary: PFS Secondary: OS, OR, AEs	Not reported	[93]
AtezoTRIBE NCT03721653	phase 2 randomised, open-label	completed	metastatic/advanced unresectable CRC	1st line	FOLFOXIRI + bevacizumab, maintenance 5-FU + Beva; FOLFOXIRI + bevacizumab + atezolizumabx8, maintenance 5-FU + Beva + Atezolizumab	N = 218 (73 + 145)	primary—PFS	mF-up 19.9 m: mPFS 11.5 vs. 13.1 m, SAEs: 26% vs. 27%%	[94]
CheckMate-142 NCT02060188	phase II	active, not recruiting	mCRC dMMR/MSI-H	previously treated cohorts	cohort 1—NIVO 3 mg/kg Q2W) cohort 2—NIVO 3 mg/kg + IPI 1 mg/kg Q3W [4 doses], then NIVO 3 mg/kg Q2W)	cohort 1 N = 74 cohort 2 N = 119	primary—ORR secondary—DCR, DOR, PFS, OS	cohort 1 mF-up 70 months: ORR 39%, PD rates 26%, mDOR NR. 48 m PFS 36%, OS 49% cohort 2 mF-up 64 m: ORR 65%, PD rates 12%, mDOR NR. 48 mPFS 54%, OS 71%	[91]
KEYNOTE-164 NCT0246019	phase II, open- label	completed	mCRC dMMR/MSI-H	pretreated cohort A ≥ 2 lines, cohort B ≥ 1 line	pembrolizumab 200 mg q3w	cohort A N = 61 cohort B N = 63	primary—ORR secondary—DOR, PFS, OS	cohort A-ORR 33%; mDOR NR; mPFS 2.3 m; mOS 31.4 m cohort B ORR 33%; mDOR NR; mPFS 4.1 m; mOS-NR	[95]
GARNET NCT02715284	phase I	completed	dMMR/ POLE mutated non-endometrial solid tumors—cohort F	previously treated	dostarlimab-gxly 500 mg q3w x 4, then 1000 mg q6w	N = 106 (N = 69 CRC tumors)	ORR	ORR in dMMR CRC—36.2%	[96]
SAMCO-PRODIGE 54 NCT 03186326	phase 2, randomised	active, not recruiting	mCRC MSI-H	second-line setting, progression after 1st line chemotherapy +/− targeted therapies	Arm A—FOLFOX or FOLFIRI +/− targeted therapy Arm B—Avelumab	N = 132	primary—PFS secondary—OS	ongoing, not reported	[97]
Keynote-016 NCT01876511	phase 2	completed	mCRC	previously treated	pembrolizumab 10 mg/kg q14d	N = 41	immune-related ORR; 20-week immune-related PFS	dMMR CRC: irORR 40%, irPFS 78%; mPFS NR, m OS NR pMMR CRC: irORR 0%, irPFS 11%; mPFS 2.2 m, mOS 5 m dMMR nonCRC: irORR 71%, irPFS 67%;	[98]
METIMMOX NCT03388190	phase 2, randomized	active, not recruiting	mCRC pMMR/MSS	1st line	control arm: 8 X FLOX Q2W experimental arm: repeat sequential 2 x FLOX and 2 nivolumab cycles (240 mg Q2W) to a total of 8 cycles	N = 80	Primary: PFS; Secondary: Safety, tolerability, ORR, DOR	mF-up 6.4 m: mPFS 5.6 m vs. 6.6 m	[99]
AVETUXIRI NCT03608046	phase 2	recruiting	mCRC pMMR/MSS, BRAF V600E wt	Pre-treated 2 cohorts A: RAS-wt, B: RAS-mt	Avelumab (anti-PD-L1) + cetuximab + irinotecan	Estimated N = 59; Interim analysis N = 23	Primary: safety, RR Secondary: DCR, PFS, OS	DCR cohort A = 60.0% and cohort B = 61.5% 6m-PFS cohort A = 40% and cohort B = 38.5% 12m-OS cohort A = 50% and cohort B = 46.2%	[100]
LCCC1632 NCT03442569	Phase 2	recruiting	mCRC pMMR/MSS KRAS/NRAS/ BRAF wild-type	1–2 prior lines	Panitumumab + nivolumab + ipilimumab	N = 56 (actual) 49 pts evaluable	Primary: ORR Secondary: PFS, OS, DOR, safety	12-wk RR = 35% mPFS = 5.7 m	[101]
CCTG CO.26 NCT02870920	Phase 2 randomized	completed	mCRC	Pre-treated, refractory to all standard systemic therapies	Durvalumab + Tremelimumab+ Best Supportive Care vs. Best Supportive Care Alone	180	Primary—OS; Secondary: PFS; ORR	mOS: 6.6 m vs. 4.1 m mPFS: 1.8 m vs. 1.9 m, NS ORR 0.8% vs. 0%	[102]
BACCI NCT02873195	Phase 2, randomized	Active, not recruiting	mCRC	previously treated, refractory	Arm I: Atezolizumab+ Bevacizumab+ Capecitabine Arm II: Pbo+ Bevacizumab+ Capecitabine	N = 133	Primary: PFS Secondary: OS, safety	In MSS pts (110/133): Non-clinically meaningfull benefit from the addition of Atezolizumab	[103]

**Table 4:** miRNAs of interest in CRC pathology involved in regulating the PD-1/PD-L1 immune checkpoint. NP—not presented.

miRNAs	MiRNA Status in CRC	Correlation of miRNA with:	miRNA Type/Molecular Mechanism Mediated by miRNA	Ref
miR-138-5p	Downregulated/reduced	Advanced clinical stage, lymph node metastases, and unfavorable OS	Tumor supressor/NP	[15]
miR-140-3p	Downregulated/reduced	Proliferation, migration and invasion as well as anti-apoptotic effects	Tumor supressor/disrupt the activation of the PI3K/AKT pathway	[114]
miR-93-5p	Downregulated/reduced	Tumor differentiation, lymphatic node metastasis, the TNM staging system and prognosis.	Tumor supressor/decrease MMP-1, MMP-2, MMP-3 expression and increase levels of IL-2, TNF-α, and IFN-γ.	[115]
miR-124	Downregulated/reduced	Cell proliferation and cell cycle activation at the G1 phase by increasing the expression of c-Myc	Tumor supressor/suppression STAT3 signaling.	[116]
miR-148a-3p	Downregulated/reduced	Tumor immune evasion in dMMR CRC	Tumor supressor/suppresive effect on cell proliferation and colony stimulation assays	[117]
miR-200a, miR-200b, miR-200c	Downregulated/reduced	Tumor invasion	Tumor supressor/mesenchymal transition of tumor cells.	[118]
miR-382-3p	Downregulated/reduced	Tumor growth and an increase in programmed cell death	Tumor supressor/NP	[119]
mikR-191-5p	Downregulated/reduced	Pathologic stage and lymphatic invasion and shorter survival	Tumor supressor/survival and tumor recurrence	[120]
miR-22	Downregulated/reduced	Immune evasion	Tumor supressor/FOSB/miR-22/BATF3/PD-L1 pathway	[122,123]
miR-497	Downregulated/reduced	Immune evasion	hsa_circ_0136666/miR-497/PD-L1 pathway	[124]
miR-214	Downregulated/reduced	Carcinogenesis and metastasis	Hipoxia: exosomal circEIF3K/miR-214 pathway	[125]

## Supplementary Materials

The following supporting information can be downloaded at: https://www.mdpi.com/article/10.3390/medicina60030397/s1, Table S1. (a) TNM staging for colon cancer, 8th ed, 2017. American Joint Committee on Cancer (AJCC). (b) Prognostic groups. TNM staging for colon cancer, 8th ed, 2017. American Joint Committee on Cancer (AJCC).
