# Supplementary material for: Molecular Subtypes, microRNAs and Immunotherapy Response in Metastatic Colorectal Cancer
Source: Medicina (Kaunas). 2024 Feb 26;60(3):397. doi: 10.3390/medicina60030397 (PMC10972200; doi:10.3390/medicina60030397)
Supplement: Supplementary file 1 [file medicina-60-00397-s001.zip › medicina-2848607-supplementary.pdf]

medicina

Review

Molecular Subtypes, microRNAs and Immunotherapy Response in Metastatic Colorectal Cancer

Alexandra Gherman [1,2], Dinu Bolundut [2], Radu Ecea [2], Loredana Balacescu [3], Sebastian Curcean [4,5], Constantin Dina [6,*], Ovidiu Balacescu [3,*] and Calin Cainap [1,2]

1 10th Department of Medical Oncology, University of Medicine and Pharmacy "Iuliu Hatieganu", 8 Victor Babes Street, 400012 Cluj-Napoca, Romania; allexandragherman@gmail.com (A.G.); calincainap2015@gmail.com (C.C.)
2 Department of Medical Oncology, The Oncology Institute "Prof. Dr. Ion Chiricuta", 34-36 Republicii Street, 400015 Cluj-Napoca, Romania; dinubolundut@yahoo.ro (D.B.); radu.ecea@gmail.com (R.E.)
3 Department of Genetics, Genomics and Experimental Pathology, The Oncology Institute "Prof. Dr. Ion Chiricuta", 400015 Cluj-Napoca, Romania; loredana_balacescu@yahoo.com
4 10th Department of Radiation Oncology, University of Medicine and Pharmacy "Iuliu Hatieganu", 8 Victor Babes Street, 400012 Cluj-Napoca, Romania; sebastian.curcean@gmail.com
5 Department of Radiation Oncology, The Oncology Institute "Prof. Dr. Ion Chiricuta", 34-36 Republicii Street, 400015 Cluj-Napoca, Romania
6 Department of Anatomy, Faculty of Medicine, Ovidius University, 124 Mamaia Boulevard, 900527 Constanta, Romania
* Correspondence: constantin.dina@gmail.com (C.D.); obalacescu@yahoo.com (O.B.)

Table S1. a. TNM staging for colon cancer, 8th ed, 2017. American Joint Committee on Cancer (AJCC) [1].

T Category	T Criteria
Tx	Primary tumor cannot be assessed
T0	No evidence of primary tumor
Tis	Carcinoma in situ, intramucosal carcinoma
T1	Tumor invades the submucosa
T2	Tumor invades the muscularis propria
T3	Tumor penetrating the muscularis propria into pericolorectal fat tissue
T4	Tumor directly invading the visceral peritoneum or adhering to other organs/structures
cT4a	Tumor penetrating visceral peritoneum
cT4b	Tumor directly invades or adheres to adjacent organs or structures
**N Category**	**N Criteria**
Nx	Regional lymph nodes cannot be assessed
N0	No regional lymph node metastasis and no tumor deposits
N1	1-3 lymph nodes metastases or tumor deposits present without lymph nodes metastases
N1a	1 regional lymph node metastases
N1b	2-3 regional lymph nodes metastases
N1c	Tumor deposits in the subserosa, mesentery or non-peritonealized,pericolic or perirectal/ mesorectal tissues; no lymph nodes metastases
N2	≥4 regional nodes metastases
N2a	4-6 regional lymph nodes metastases
N2b	≥7 regional lymph nodes metastases
**M Category**	**M Criteria**
M0	No distant metastasis
M1	Metastasis to one or more distant sites or organs
M1a	Metastasis to one site/ organ, no peritoneal metastasis

M1b	Metastasis to ≥2 sites/ organs, no peritoneal metastasis
M1c	Peritoneal metastasis +/- other sites of metastasis

Table S1. b. AJCC prognostic groups. TNM staging for colon cancer, 8th ed, 2017. American Joint Committee on Cancer (AJCC) [1].

	**T**	**N**	**M**
Stage O	Tis	N0	M0
Stage I	T1, T2	N0	M0
Stage IIA	T3	N0	M0
Stage IIB	T4a	N0	M0
Stage IIC	T4b	N0	M0
Stage IIIA	T1, T2	N1/N1c	M0
	T1	N2a	M0
Stage IIIB	T3, T4a	N1/N1c	M0
	T2, T3	N2a	M0
	T1, T2	N2b	M0
Stage IIIC	T4a	N2a	M0
	T3-T4a	N2b	M0
	T4b	N1, N2	M0
Stage IVA	Any T	Any N	M1a
Stage IVB	Any T	Any N	M1b
Stage IVC	Any T	Any N	M1c

Reference

1. Amin, M.B.; Edge, S.; Greene, F.; Byrd, D.R.; Brookland, R.K.; Washington, M.K.; Gershenwald, J.E.; Compton, C.C.; Hess, K.R.; Sullivan, D.C.; et al. *AJCC Cancer Staging Manual*, 8th ed.; Springer: Cham, Switzerland, 2017.
